# Editorial: Exploring the psychology of vocational education: From the perspective of literacy promotion

**DOI:** 10.3389/fpsyg.2023.1167176

**Published:** 2023-03-14

**Authors:** Jian-Hong Ye, Mei-Yen Chen, Yung-Wei Hao

**Affiliations:** ^1^Faculty of Education, Beijing Normal University, Beijing, China; ^2^National Institute of Vocational Education, Beijing Normal University, Beijing, China; ^3^Graduate Institute of Sport, Leisure and Hospitality Management, National Taiwan Normal University, Taipei, Taiwan; ^4^Graduate Institute of Curriculum and Instruction, National Taiwan Normal University, Taipei, Taiwan

**Keywords:** psychology of vocational education, vocational education, vocational probation, vocational preparation, continuing vocational education

Vocational education helps students develop or enhance their knowledge, skills and abilities for a particular occupation. As a result, vocational education plays an important role in the economic and social development of countries (and regions) around the world. It has also made an important contribution to educational equity. The development of quality and sustainable vocational education has become an issue of concern for governments, educational institutions, educators and researchers. In the past, many experts have been thinking about how to develop policies to help promote vocational education. However, in addition to national goals, industry needs and expert opinions, the education policy system also needs to take into account the views and opinions of the stakeholders in order to implement the policy effectively.

The psychology of vocational education is broad in scope, because it encompasses stages of vocational probation, vocational preparation and continuing vocational education, from K-12 education to higher education and to lifelong education. Consequently, the issue of how to improve the quality and learning outcomes of vocational education has become an important topic in education. From the theoretical perspective of educational psychology, psychological and cognitive factors can be used to facilitate students' learning performance or to enhance teachers' teaching effectiveness. It can also help teachers in vocational schools better understand and develop students' vocational literacy in a more in-depth way, so that they can better respond to the challenges of their work.

The psychology of vocational education can be based on a cross-disciplinary theoretical approach to facilitate educational policy implementation, educational management, curriculum development, instructional design, apprenticeship, workplace learning, career counselling, skills training, career preparation, workplace learning, and so forth. The importance of the psychology of vocational education is therefore self-evident.

The study of the psychology of vocational education contributes to the development of vocational and technical personnel and the understanding of individual differences. It is more helpful to identify the effects or problems from the perspectives of the persons involved and to understand the reality of the situation. There is a wide range of specific Research Topics in the psychology of vocational education. Examples include: academic achievement (e.g., Liu Y.-B. et al.; Peng et al.), career adaptation (e.g., Chuang et al.; Jiang et al.; Wang et al.; Xia and Wang), career exploration (e.g., Liu X. et al.), creativity (e.g., Niu and Wu), critical thinking (e.g., Sartori et al.), discrimination perception (e.g., Liu Y.-B. et al.), education climate (e.g., Xue et al.), employability (e.g., Bai et al.), entrepreneurship education (e.g., Niu et al.), learning engagement (e.g., Jiang et al.; Lu et al.), learning methods (e.g., Hong et al.; Zhou), learning motivation (e.g., Chang et al.; Chuang et al.), learning readiness (e.g., Loock et al.), online learning (e.g., Niu and Wu; Ye et al.), postgraduate education (e.g., Chang et al.), professional literacy (e.g., Fan), prosocial behavior (e.g., Su and Wang), self-efficacy (e.g., Chen and Ma; Chuang et al.; Niu et al.), skills training (e.g., Thianthai and Sutamchai), social support (e.g., Chen and Ma; Jiang et al.; Liu Y.-B. et al.; Peng et al.; Su and Wang), and teaching methods (e.g., Li et al.). As can be seen from the above, there is a wide range of research issues in the psychology of vocational education.

In addition, the dramatic impact of the COVID-19 pandemic on education systems around the world has given us insights into the impact of context in educational research. From the perspective of the psychology of vocational education, we have also gained a better understanding of the learning difficulties encountered by vocational education students and the teaching constraints of teachers when learning online. While it is important to understand successful stories, the reasons why they fail or succeed also need to be explored in order for us to understand the reasons. The results of these analyses can also help us understand how to effectively improve the design of online teaching and learning. In the post-epidemic era, vocational education is entering a new phase. Researchers, teachers and government agencies will be considering how to compensate for the impact of the COVID-19 epidemic on vocational education. This highlights the need for more research on teaching and learning to inform us of how up-to-date vocational education is.

Furthermore, because of the COVID-19 epidemic, positive psychology has gained more attention in vocational education. Therefore, future research could be conducted on the role of helping people understand positive and negative emotions, career choices and career adaptation in the process of career development and construction, within the framework of occupational emotion theory. We can also use more positive psychology-related theories and variables to help us understand the psychology of vocational education.

It is clear from the above that, given the specificity and importance of the psychology of vocational education, interdisciplinary research methods, theoretical foundations and statistical models are needed to help us understand it more comprehensively. As time changes, new variables and localized variables need to be proposed to explain more current situations. In addition, the development of research on the psychology of vocational education needs to take into account the characteristics of the localization, global trends and educational policy directions, as well as to extend the scope of the psychology of vocational education to different cultures, groups and contexts. It is also possible to expand the psychology of vocational education in different cultural groups and contextual contexts. The need for education encompasses many dimensions, from the Macrocosm, to the mesocosm, to the microcosm—all need to be considered.

Of course, the research themes in the psychology of vocational education are not limited to those mentioned above. We are also looking forward to a wider range of Research Topics to help us understand the situation of vocational education. Although the formal system of vocational education is only a few hundred years old, the concept of skill training has been around since the creation of civilization. The development of vocational talent has also been around for thousands of years. Therefore, it needs to be paid with more attention and importance.

## Author contributions

J-HY and M-YC: concept, design, and drafting of the manuscript. J-HY and Y-WH: critical revision of the manuscript. All authors contributed to the article and approved the submitted version.

